# Prophylaxis Against Thromboembolic Events During Chemotherapy for Germ Cell Cancer

**DOI:** 10.3389/fonc.2021.724682

**Published:** 2021-10-07

**Authors:** Xiaosong Meng, Murtaza Ahmed, Kevin D. Courtney, Waddah Arafat, Ibrahim Ibrahim, Vitaly Margulis, Craig Nichols, Aditya Bagrodia

**Affiliations:** ^1^Department of Urology, University of Texas Southwestern Medical Center, Dallas, TX, United States; ^2^School of Medicine, University of Texas Southwestern Medical Center, Dallas, TX, United States; ^3^Department of Internal Medicine, University of Texas Southwestern Medical Center, Dallas, TX, United States; ^4^Testicular Cancer Commons, Portland, OR, United States; ^5^Department of Urology, University of California San Diego, San Diego, CA, United States

**Keywords:** germ cell tumor, thromboembolic event, chemotherapy, anticoagulation, cisplatin, khorana risk score, solid tumor

## Abstract

**Introduction:**

Patients with advanced germ cell tumors (GCT) receiving cisplatin-based chemotherapy have high rates of thromboembolic events (TEE) which can negatively affect their overall survival. While primary TEE prophylaxis during chemotherapy may prevent these events, it is unclear which patients will benefit in this setting.

**Materials and Methods:**

A review of PubMed/Medline was conducted in December 2020 and all pertinent articles were evaluated for relevancy and quality of data for inclusion in the review.

**Results:**

Studies on patients receiving initial cisplatin-based chemotherapy for advanced GCT have reported up to a 19% rate of TEE. This high rate may be associated with multiple factors including retroperitoneal lymphadenopathy, advanced clinical stage, high risk Khorana scores and presence of a central line. Large phase III clinical trials have demonstrated the benefit of low-molecular-weight-heparin and direct oral anticoagulants for primary prophylaxis and against recurrent TEE. However, primary prophylaxis is currently underutilized with GCT patients starting chemotherapy.

**Conclusion:**

Precise models to predict TEE risk and consideration of anticoagulation are difficult to develop owing to the relatively uncommon nature of GCT and lack of representation in primary TEE prophylaxis clinical trials. Despite these limitations, we believe that the benefits of prophylactic anticoagulation outweigh the risk of major bleeding in select GCT patients with higher risk of TEE. We have developed a simple algorithm to help guide TEE prophylaxis selection based on patient factors and route of chemotherapy administration. Given the high rate of TEE in GCT patients, we believe better utilization of primary prophylaxis in patient starting cisplatin-based chemotherapy will have clinical benefit.

## Introduction

Testicular cancer is the most common solid tumor in men between the ages of 20 and 34 years, with an estimated 9610 new cases in the United States for 2020 and 5-year relative survival rate of 95% (1, 2). Combination cisplatin-based chemotherapy for disseminated germ cell tumors (GCT), first described in the late 1970s (3), has led to dramatically improved survival and is the standard of care systemic therapy. Given highly effective curative therapy, an important area of attention should be on improving the sequelae of curative treatment in this population of young patients and their post-treatment quality of life (4). There are a number of potential established side effects of cisplatin-based chemotherapy such as neuropathy, ototoxicity, secondary malignancy, hormonal changes, infertility, cardiovascular, pulmonary, renal toxicity, and a number of hematological abnormalities such as myelosuppression and an increased thromboembolic risk (5). An elevated risk of thromboembolic events (TEE) is inherent to cytotoxic chemotherapy and most commonly include venous thromboembolism (VTE) such as deep venous thrombosis (DVT) and pulmonary embolus (PE). VTE occur frequently and are associated with significant morbidity and downstream consequences in this population of young patients.

There are multiple underlying causes for increased TEE in cancer: 1) the inherent prothrombotic state of malignancy 2) patient factors such as decreased mobility from hospitalization, vascular disease and underlying coagulation disorders, and 3) treatment related factors due to central lines, surgery, radiation or chemotherapy (6–9). These factors combined contribute to a four to seven-fold increase in risk of TEE compared to patients without malignancy, with certain malignancies, treatments and underlying co-morbidities increasing this risk further (6, 8, 9). In a population-based case-control study, patients with malignancy treated with chemotherapy had a 6.5-fold increased risk of TEE compared to a 4-fold increased risk of TEE from malignancy alone (6). Even more concerning, patients diagnosed with TEE during the first year of follow-up have decreased overall survival compared to those without evidence of TEE for all cancer types (10, 11). This is evident in the inpatient setting, where a study of hospitalized neutropenic cancer patients found up to a 5-fold risk of in-hospital mortality (12). In the outpatient setting, a prospective study examining the cause of death in cancer patients starting a new chemotherapy regimen found that TEE (9.2%) and infection (9.2%) were leading non-cancer causes of death in this patient population (13). Overall, cancer patients with TEE undergo more hospitalizations, have a higher rate of metastatic disease and worse overall survival compared to cancer patients without TEE (11).

Multiple retrospective studies have demonstrated an increased risk of TEE in patients with GCT treated with chemotherapy. Piketty reported a 19% incidence of TEE in GCT patients receiving cisplatin-based chemotherapy, which was significantly higher than non-GCT age and sex matched controls receiving cisplatin-based chemotherapy (6%) (14). Paffenholz found similar rates of TEE in their multi-center observational cohort study of 255 patients receiving cisplatin-based chemotherapy, with 19% overall TEE (15). In the largest study to date involving 1135 patients with metastatic GCT receiving cisplatin-based chemotherapy at 22 centers, Tran reported a TEE rate of 13% (16).

The Khorana predictive model for chemotherapy-associated thrombosis, first introduced in 2008, is perhaps the best-known risk stratification tool to help guide physicians on which cancer patients need further intervention (17). This model assigns points to the site of cancer, obesity, and three pre-chemotherapy laboratory parameters or use of erythropoiesis-stimulating agents. Patients with a sum Khorana score of 0 are considered low risk, those with 1 or 2 points are considered intermediate risk and those with 3 or more points are considered high risk of developing symptomatic TEE over the next few months (17). The American Society of Clinical Oncology (ASCO) endorses the use of the Khorana score as a method for identifying which ambulatory cancer patients should be considered for thromboprophylaxis (18).

However, given the relative rarity of testicular cancer, very few GCT patients were represented in the original models. As such, models such as the Khorana score may not be applicable to this young healthy population. Additionally, this model does not account for potential anatomic changes due to retroperitoneal lymphadenopathy around the great vessels from metastatic GCT. Given that one in five patients receiving initial chemotherapy for advanced GCT may develop a TEE (14, 15, 19), we set out to review the contributing factors to the development of TEE in GCT patients receiving cisplatin-based chemotherapy, the current options and utilization of anticoagulation in other malignancies and the rational for applying this to testis cancer patients.

## Methods

**Figure 1:**
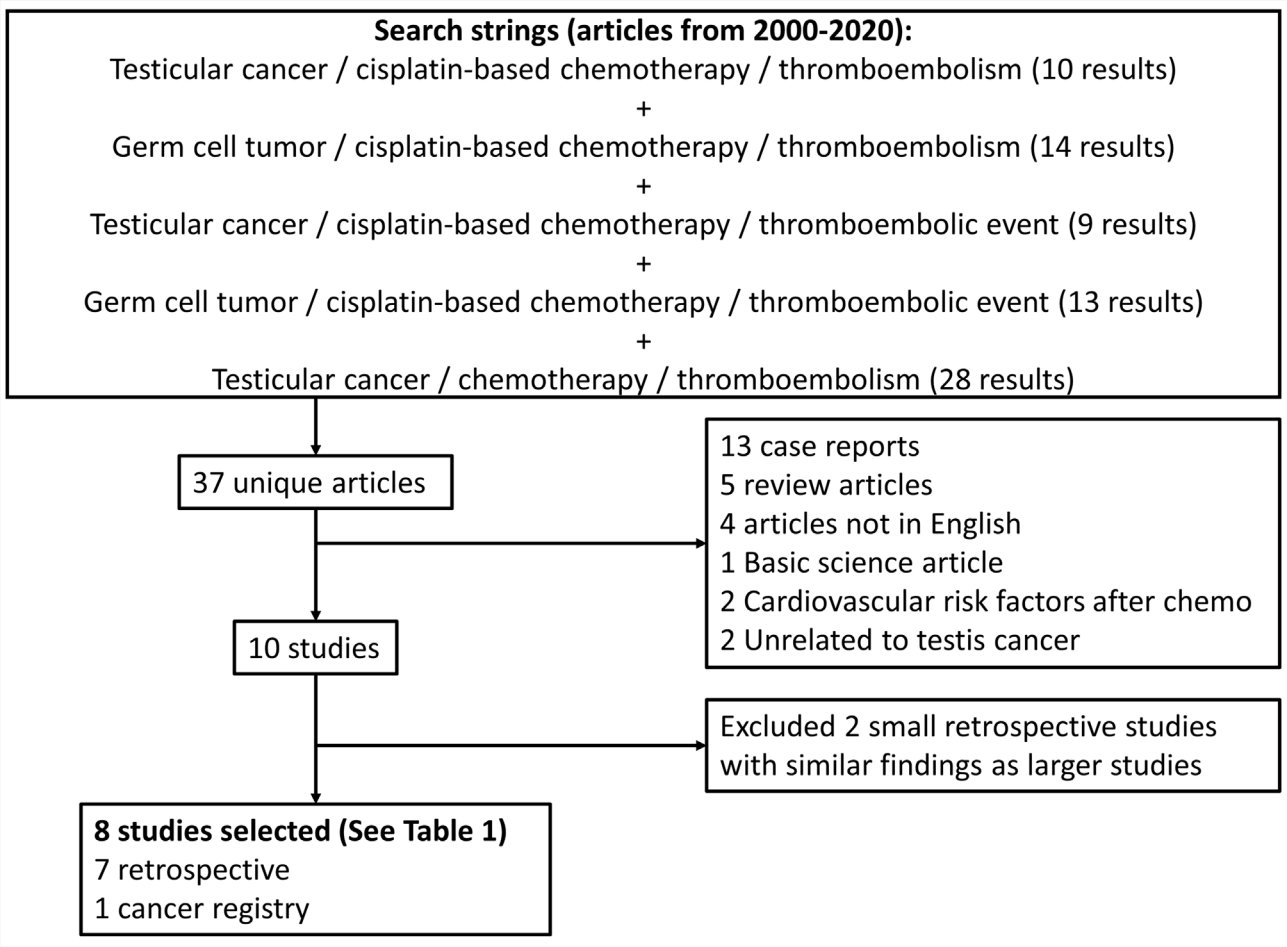
Study flowchart.

We performed a review of PubMed/Medline in December 2020 for articles from 2000-2020 using a combination of search strings including testicular cancer or germ cell tumor plus thromboembolism or thromboembolic event plus cisplatin-based chemotherapy (**Figure 1**). Further articles looking at recent data on prophylactic anticoagulation and anticoagulation therapies for venous thromboembolism were also reviewed. All study designs were accepted except for case reports. We limited the analyzed studies to those published in the English language, original studies, and meta-analyses. Pertinent articles were reviewed for relevancy and quality of data for inclusion in the review.

## Results

### Pathophysiology – Risks of TEE With Cisplatin-Based Chemotherapy

Although the pathogenesis of cisplatin-induced thrombogenicity is not fully elucidated, potential mechanisms such as endothelial injury, platelet activation and upregulation of prothrombotic factors likely contribute to its thrombogenic effects (20–22). In a large retrospective analysis of 932 patients receiving cisplatin-based chemotherapy for any malignancy, 18% of patients were found to have a TEE during treatment or within four weeks of completing treatment, with DVT and PE accounting for almost 90% of the events (23). In a larger meta-analysis involving 8216 patients treated with a variety of chemotherapy regimens for different solid tumors from 38 randomized controlled trials, patients who received cisplatin-based chemotherapy had a 1.67-fold increased likelihood of experiencing a TEE compared to those not receiving cisplatin-based chemotherapy (24).

### Clinical Presentation

Patients with metastatic GCT can either present with TEE at cancer diagnosis, develop TEE during the course of chemotherapy or develop TEEs shortly after completing chemotherapy. Out of these three groups, patients most commonly develop TEE during the course of chemotherapy. Of the 657 consecutive GCT patients at the Medical University of Graz over a 13-year period, only 3 patients had TEE at cancer diagnosis, while 34 patients experienced a TEE within the first year (19). Of these 34 patients, 4 patients developed a TEE during follow-up without exposure to chemotherapy while the remaining 30 (88%) had at least 2 cycles of chemotherapy. Likewise, in the Piketty study, of the 29 out of 177 patients who developed their first TEE, 72% developed their TEE during chemotherapy while the remaining 28% developed a TEE within 6 months of completing chemotherapy (14). In a multi-center study by the global germ cell cancer group (G3) composed of 1135 patients with a total of 150 TEEs, 35% were diagnosed with TEE immediately prior to initiation of chemotherapy, 52% during chemotherapy and 12% immediately following chemotherapy. An additional 2 patients were diagnosed with TEE in the postoperative setting after completing chemotherapy (16). However, it should be noted that patients who present with significant retroperitoneal lymphadenopathy may have a higher incidence of TEEs at diagnosis. Out of the 24 patients who had TEEs prior to chemotherapy in the Srikanthan study, 46% of these had retroperitoneal lymphadenopathy that was more than 5 cm (25).

The range of symptomatic TEEs varied between 55%-65% among different studies, but deaths from TEE were rare (16, 19). The majority of TEEs were composed of DVT and PEs, with PE alone (59%), DVT alone (24%) and DVT and PE (15%) in one cohort (19). Similar to the Bezan study, the majority of TEEs were comprised of DVTs and PEs (86%), with only 1 arterial thrombosis (14). In the G3 study, out of 150 TEEs, 30% were abdominal DVT, 7% upper extremity DVT, 18% lower extremity DVT, 28% PE and 14% were vascular access device associated (16).

### Assessment – Risk Factors for TEE in Patients With Germ Cell Tumors

While there are likely multiple causes of increased TEE rates in GCT patients, patients with large retroperitoneal lymph nodes (RPLN) may be particularly at risk due to mass effect on the major blood vessels, causing venous stasis. In the Tran study discussed above, patients with RPLN >3.5cm had significantly higher risk of TEE compared to those with smaller RPLN (22% versus 8%, OR 3.0) (16). These findings corroborate the results of a prior study that found TEE was associated with RPLN >5 cm (OR 5.26). Additionally, the risk of TEE is also increased in patients with higher clinical stages as demonstrated using a predictive, externally validated model with clinical stage IIC patients with 12-month incidence of 11-14% and clinical stage IIIA-C patients with 19-21% incidence of TEE (19).

Central venous access has also been shown to be a significant risk factor for TEE. In their multi-center observational cohort study of GCT patients, Paffenholz reported that central venous access (CVA) on multivariate analysis showed an increased risk for TEE with an odds ratio (OR) of 3.5 (15). In their multi-institutional retrospective analysis of patients receiving first-line platinum-based chemotherapy, Fankhauser et al. reported that a venous access device was the one risk factor for development of TEE during or after chemotherapy (26). This association between TEE and CVA is also reflected in other cancers, with a OR of 3.4 with the presence of CVA device in patients receiving pre-operative chemotherapy for esophagogastric adenocarcinoma (27). These studies demonstrate that for patients receiving chemotherapy regimens such as BEP/EP that do not require central venous access for administration, the convenience of central venous access needs to be carefully weighed against the significantly increased risk of TEE. For patients who need ifosfamide-containing regimens that require central venous access, the inclusion of TEE prophylaxis should be included in the risk assessment model. Additionally, other predictive factors of TEE in patients with GCT receiving cisplatin-based chemotherapy include elevated serum lactate dehydrogenase, high body surface area, febrile neutropenia, increasing number of cycles of chemotherapy and Khorana score ≥3 (14–16, 28) (**Table 1**).

**Table 1:** Risk factors for thromboembolic events in germ cell tumor studies.

Author	Time frame	Number of patients	Follow-up window	Stage	Regimen	Rate of TEE	Location of TEE	Timing of TEE in relation to chemotherapy	Risk factors
Piketty et al.	1992 - 1998	100	6 mo	I: 18% II: 42% III: 40%	BEP, EP, CISCA-VB	19%	DVT: 84% SVT: 11% Arterial: 5%	During: 74% After: 26%	Weight > 70kg: RR 7.4 Elevated LDH: RR 5.9
Gizzi et al.	2001-2014	279	6 mo	I: 17% II: 42% III: 37%	BEP, EP, paclitaxel-BEP	14% *43% of cohort received LMWH ppx	DVT: 69% SVT: 26% Arterial: 5%	Prior: 16 pts * excluded from study During: NR After: NR	Elevated LDH: OR 3.41 No adenopathy: OR 0.30
Paffenholz et al.	2003-2018	252	6 mo	I: 28% II: 36% III: 36%	BEP, TIP, PEI	19%	DVT: 94% Arterial: 6%	During: 57% After: 4% Unknown: 39%	Clinical stage ≥ IIC: OR 2.26 Elevated LDH: OR 2.16 Central line: OR 3.47 Febrile Neutropenia: OR 2.97
Bezan et al.	2000-2013	657	12 mo	I: 74% II: 15% III: 11%	56% no chemo 44% chemo, regimen NR	5.2% * 3% of cohort received primary ppx	DVT only: 23% PE only: 59% DVT/PE: 15% Visceral: 3%	Prior: 3 pts * excluded from study During: NR After: NR	Clinical Stage IS, IIA-C: SHR 1.97 Clinical stage IIIA-C: SHR 4.87 >5cm RPLN: SHR 3.29 Inter/Poor risk: SHR 2.61 Elevated LDH: SHR 2.37 Khorana score ≥ 2: SHR 2.22
Tran et al.	2000-2014	1135	NR	I: 5% II: 42% III: 51%	BEP, EP, VIP	10%	DVT: 55% PE: 28% Vascular access device: 14%	Prior: 35% During: 52% After: 12%	>3.5cm RPLN: OR 1.81 RP primary: OR 3.30 Khorana score ≥ 3: OR 2.62 Vascular access device: OR 2.66
Srikanthan et al.	2000-2010	216	3 mo	NR	Cisplastin-based	10% * 8% of cohort received LMWH ppx	NR	Prior: 8% During: 10%	Hospitalization: OR 4.24 Inter/Poor risk: OR 3.76 >5cm RPLN: OR 5.26 Khorana score ≥ 3: OR 11.8
Fankhauser et al.	1998-2015	1199	NR	NR	BEP (76%) EP (7%) VIP (2%) TIP (<1%)	11%	DVT: 56% PE: 28%	Prior: 40% During: 46% After 13%	Venous access device: OR 1.8
Robinson et al.	2000-2010	2650	5 years	NR	BEP, EP	8%	NR	Prior: 13% During: 62% After: 25%	Four cycles of chemo: OR 3.91 Three cycles of chemo: OR 1.63

BEP, Bleomycin, etoposide, cisplatin.

EP, Etoposide, cisplatin.

CISCA-VB, cisplatin, cyclophosphamide, doxorubicin and vinblastine/bleomycin.

TIP, Paclitaxel, ifosfamide, cisplatin.

VIP, Etoposide, ifosfamide, cisplatin.

PEI, Modified cisplatin, etoposide, ifosfamide.

DVT, Deep vein thrombosis.

SVT, Superficial vein thrombosis.

PE, Pulmonary embolism.

NR, Not reported.

RR, Relative risk.

OR, Odds ratio

SHR, Subhazard ratio.

RP, Retroperitoneal.

RPLN, Retroperitoneal lymph node.

LDH, lactate dehydrogenase.

*Correlates to a note for that field.

Given the high rate of TEE in patients with advanced GCT receiving platinum-based chemotherapy, chemoprophylaxis against TEE has been proposed by many to mitigate this adverse clinical event (14, 19, 23, 29). This is indeed the case in Germany, where a survey evaluating prophylactic anticoagulation with LMWH found that it was administered in 94% of the clinics in the German Testicular Cancer Study Group, with another 33% continuing anticoagulation after the completion of chemotherapy for 2 to 24 weeks. However, given no clear guidelines on TEE prophylaxis in GCT patients in Germany, there was significant variations in the duration and dosage of anticoagulation (30). This is in contrast to the low rate of prophylactic anticoagulation usage seen in the G3 study, were only 7% of the cohort received anticoagulation for longer than 7 days (16). Similarly, in the US, the National Comprehensive Cancer Network (NCCN) guidelines on Cancer-Associated Venous Thromboembolic Disease, no specific guidelines have been developed for GCT, but that discussions of risks/benefits of TEE prophylaxis should be performed in patients who are high risk based on Khorana risk assessment (31). However, given certain limitations in applicability of the Khorana score in this population, a consistently high rate of TEE in GCT patients receiving cisplatin-based chemotherapy, and inferior outcomes in patients who develop TEE, we strongly believe that this population of patients warrant consideration of prophylactic anticoagulation during chemotherapy.

## Discussion

### Evolution of Options for Anticoagulant Therapy in Cancer Patients

LMWH represented the standard of care for prevention of malignancy-associated TEEs after multiple large-scale trials and meta-analyses demonstrated superiority over warfarin (32–36). A newer class of agents, the direct oral anticoagulants (DOACs), were developed as an alternative method that addresses the need for subcutaneous injections with LMWH and numerous disadvantages of warfarin (37). DOACs have since replaced warfarin as the standard of care for treatment of TEE in the general population based on multiple large, randomized trials demonstrating their non-inferiority to warfarin for prevention of recurrent TEEs (38–41). A meta-analysis of six phase III DOAC versus warfarin trials found that DOACs may have better efficacy over warfarin [Relative risk (RR) 0.57] without increasing the risk of major bleeding for patients with malignancy as well (42). Three multi-center randomized controlled trials have also directly compared DOACs against LMWH in patients with active malignancy (43–45). All three trials demonstrated superiority or non-inferiority of DOACS to LMWH with modest increases in bleeding events, given physicians multiple anticoagulation modalities for patients with cancer.

### Primary Prophylaxis Against Thromboembolic Events

Two large, randomized trials, SAVE-ONCO and PROTECHT, have compared the use of LMWH versus placebo in patients with a variety of malignancies for primary prophylaxis of cancer-associated TEE (46, 47). Both trials demonstrated significant decreases in VTE events in the treatment arm with no difference in clinically relevant or major bleeding between the two groups.

Two large, randomized trials have also been performed with DOAC in the primary prophylaxis setting. The CASSINI trial assessed the efficacy and safety of rivaroxaban compared to placebo in ambulatory cancer patients initiating chemotherapy with Khorana score of 2 or more (48). Over the study period of 6 months, 6% of patients receiving rivaroxaban and 8.8% of patients receiving placebo experienced either a DVT, PE or death from TEE (HR 0.66, p=0.10). However, there was a statistically significant difference in TEE incidence in patients receiving rivaroxaban versus placebo during the intervention period (2.6% with rivaroxaban versus 6.4% in placebo, HR 0.4). Major bleeding occurred in 2% of patients in the rivaroxaban group and 1% in the placebo group (48). The AVERT trial compared primary prophylaxis with apixaban versus placebo in patients with Khorana Score >2 and found that patients receiving apixaban had a lower risk of TEE compared with placebo (1% with apixaban versus 7.3% with placebo, HR 0.14, p<0.001) but the risk of major bleeding was increased with apixaban (HR 2.0, p=0.046) (49). Of note, neither the LWMH nor DOAC trials appeared to include any significant number of testicular cancer patients.

### Primary Prophylaxis in Advanced Germ Cell Tumor Patients

In the most recent TEE Prophylaxis Guideline update, the American Society of Clinical Oncology (ASCO) recommended against routine pharmacologic thromboprophylaxis in ambulatory patients, except in high-risk patients with Khorana score of 2 or higher prior to starting systemic chemotherapy (18). For these patients, the ASCO guidelines recommend apixaban, rivaroxaban or LMWH if the patient has no significant risk factors for bleeding and after a discussion with the patient about the relative risks and benefits of starting prophylaxis (18). Given that GCT patients have a baseline Khorana score of 1 due to cancer type, patients with high BMI (≥35 kg/m^2^) or laboratory abnormalities in platelet count, hemoglobin or leukocyte count would fall into this higher risk category where primary prophylaxis could be considered.

Several specific factors in GCT patients should be considered when deciding on anticoagulation. Patients with pure or predominantly choriocarcinoma have an increased risk of tumoral hemorrhage, attributed to the biological behavior of choriocarcinoma cells which are known to invade and erode blood vessels (50). Additionally, patients with very high choriogonadotropin levels and numerous pulmonary metastases can be at risk for a rare condition termed choriocarcinoma syndrome when starting systemic chemotherapy, characterized by acute respiratory syndrome and hemorrhage from metastatic sites with a high mortality rate (51). While no data exists for anticoagulation usage in these patients, it may be prudent to avoid anticoagulation in these patients given their higher risk of adverse hemorrhagic complications.

The other category of patients requiring special considerations are those with brain metastases. While patients with brain metastases are not well represented, one recent study in patients with glioblastoma or brain metastases with atrial fibrillation did not have increased risks of intracranial hemorrhage due to anticoagulation in a series of 104 patients from 2005-2017 (52). Additionally, in the prophylaxis of VTE in patients with cancer review, Farge et al. recommended that a brain tumor was not a contraindication for anticoagulation for established VTE (Grade 2C) and that LMWH was preferred (53). Additionally, for patients undergoing neurosurgery for brain tumors, prospective randomized studies found that LMWH and UFH decreased risk of post-operative VTE by 50% without major bleeding risk but did double the minor bleeding risk compared to no treatment. Extrapolating from this data, patients with brain metastases should not automatically be excluded from prophylactic anticoagulation but be counseled on the risks and benefits of starting anticoagulation with a brain metastasis.

In terms of anticoagulation selection, while all of the DOACs undergo renal elimination, there are variations in the degree of renal metabolism for each DOAC (37). Of these, dabigatran has the highest renal elimination with 80% compared to only 25% for apixaban, an important consideration in selection of DOAC therapy given the known renal toxicities of cisplatin (5, 37). Additional pharmacologic properties of DOACs that warrant consideration include their dependence on gastrointestinal tract absorption. Patients with post-operative nausea/vomiting or emesis from chemotherapy may affect the absorption of DOACs and hence be better managed with LMWH, as well as patients with prior gastrointestinal tract surgery (54). Finally, DOACs have important drug-drug interactions with several chemotherapeutic agents commonly employed for GCT patients, including etoposide (bleomycin, etoposide, cisplatin regimen – BEP), ifosfamide (etoposide, ifosfamide, cisplatin regimen – VIP), paclitaxel (paclitaxel, ifosfamide, mesna, cisplatin regimen – TIP) and anthracyclines such as doxorubicin (37). Interactions between these chemotherapeutic agents, DOACs and the CYP3A4 enzyme and P-glycoprotein can alter the level of anticoagulation of DOACs and predispose patients to bleeding or thrombotic complications (37).

### Limitations to the Khorana Score for GCT Patients

The risk of TEE based on Khorana score was further evaluated though a systematic review and meta-analysis using studies from 2008 to 2018, forming a cohort of 34,555 ambulatory cancer patients with 81% of the cohort having 6 months of follow-up (55). In the first six months, ambulatory cancer patients with a low-risk Khorana score (0 points) had an 5% incidence of TEE, intermediate-risk Khorana score (1 - 2 points) patients had an 6.6% incidence and high-risk Khorana score (3 or higher) had an 11% incidence (55). Surprisingly, in the entire cohort, 76.6% of patients who developed a TEE in the first 6 months were in the low or intermediate risk groups (55). When looking at studies in the meta-analysis that focused on testicular cancer only, patients with intermediate-risk Khorana score had a 5.9% incidence and patients with high-risk Khorana score had a 22.3% incidence (55). The higher incidence of TEE in the high-risk group for GCT patients over other malignancies lends more support to the notion that selective use of primary prophylaxis in GCT patients starting cisplatin-based chemotherapy is warranted. However, as the original Khorana predictive model only included 17 patients with GCT, the Khorana score alone may not be the best representation of this cohort (25). One study in metastatic GCT patients found that large RPLN (>5cm) had higher discriminatory accuracy than high-risk Khorana score (≥3) in predicting TEE (25). Additionally, using the Graz cohort to evaluate one-year risk of TEE, multivariable competing risk regression adjusting for chemotherapy found a higher sub hazard ratio (45) for patients with clinical tumor stage IIIA-IIIC (SHR 4.89), >5cm RPLN (SHR 3.29), intermediate and poor IGCCCG risk disease (SHR 2.61) and elevated LDH (SHR 2.37) than patients with Khorana score ≥ 2 (SHR 2.22) (19). These factors demonstrate that while patients with a high-risk Khorana score should be strongly considered for primary prophylaxis, additional GCT specific factors may warrant consideration even with a low risk Khorana score.

### Duration of Prophylaxis and Peri-Operative Considerations for Patients on Anticoagulation

There is a lack of consensus on the duration necessary for prophylactic anticoagulation in GCT patients. In the G3 study, only 7% of the cohort received prophylactic anticoagulation for more than 1 week compared to the 33% of clinics in Germany that continued anticoagulation for 2-6 weeks after the end of chemotherapy (16, 30). While there is no specific recommendations for GCT, the NCCN guides do advocate for consideration of anticoagulant prophylaxis for up to 6 months in patients with Khorana score ≥ 2 (31). In the absence of more concrete data on the duration of prophylaxis, we believe that patients should be started and maintained on anticoagulation throughout their entire chemotherapy regimen. Given data suggesting that TEEs rarely occur after conclusion of chemotherapy (26), in our opinion, anticoagulation prophylaxis can likely be stopped in the majority of patients unless multiple high-risk factors are still present.

For GCT patients who are on prophylactic anticoagulation prior to surgery, considerations for stopping anticoagulation and bridging therapy should follow existing guidelines (31). As patients undergoing retroperitoneal lymph node dissection (RPLND) have a very high bleeding risk and moderate to high-risk TEE risk category, for patients on prophylactic LMWH, their last dose should be 24 hours prior to surgery. Generally, for patients on apixaban, dabigatran or edoxaban, their last dose should be around 2.5 to 4 days prior to surgery. Patients on fondaparinux need their last dose held around 4 to 5 days given the longer half-life while patients on rivaroxaban only need it held around 1.5 to 2.5 days prior (31). Patients who have renal impairment generally need their last dose held a day or two sooner but should be individualized to the patient and their particular DOAC, given variations in renal metabolism among DOACs (37). Post-operatively, NCCN guidelines recommend restarting patients on LMWH post-procedural with the first prophylactic dose around 24 hours with transition to DOACs around 7 days (31). While restarting DOACs at an earlier time point is possible given FDA approval of specific reversal agents for DOACs (idarucizumab for dabigatran, andexanet alfa for apixaban and rivaroxaban) and hemostatic agents such as 4 factor prothrombin complex concentrates, these factors should be weighed against possible decreased post-operative gastrointestinal absorption due to nausea/vomiting, renal dysfunction and the cost/availability of expensive reversal agents (56–58). Additionally, all of the reversal agents and hemostatic agents are prothrombotic to varying degrees, and risk of bleeding and patient convenience factors from a DOAC over LMWH need to be carefully balanced in the post-operative window.

## Recommendation

**Figure 2:**
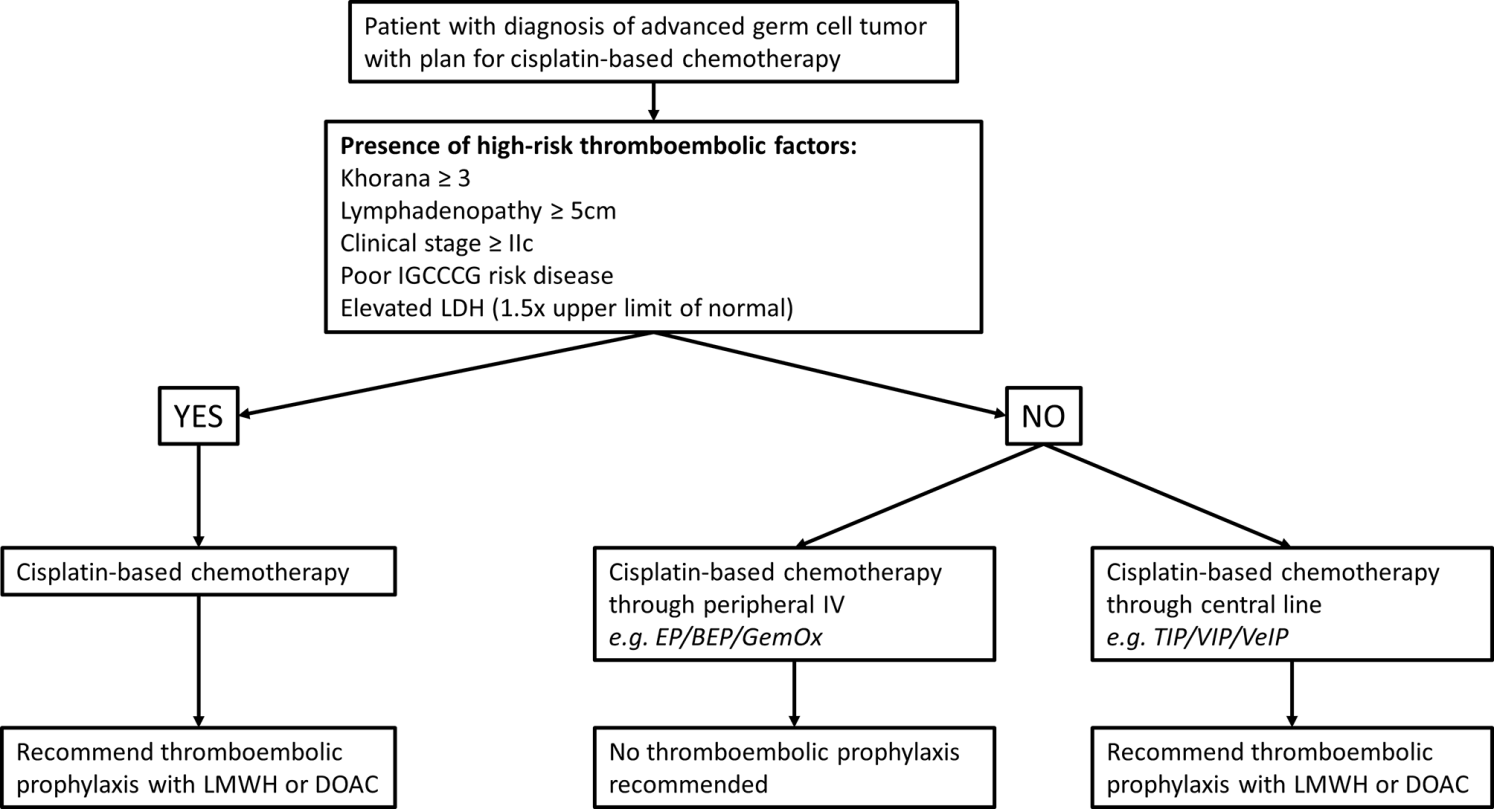
Thromboembolic event prophylaxis flowchart.

TEE are common and potentially very serious complications of germ cell tumors and cisplatin-based chemotherapy management. Cisplatin, the mainstay drug in GCT combination chemotherapy is felt to have relatively high thrombogenic potential and the natural anatomic distribution of regional and metastatic disease add risk through the common involvement of the retroperitoneal and superior vena cava. Precise models to predict TEE risk and consideration of prophylactic anticoagulation are difficult to develop owing to the relatively uncommon nature of germ cell tumors and even fewer who ever require chemotherapy. Common models such as the Khorana models are not entirely applicable to this young healthy population and very few germ cell tumor patients were represented in the original models. Despite these limitations, we believe that the benefits of prophylactic anticoagulation outweigh the risk of major bleeding in select patients due to their higher risk of TEE due to disease biology and treatment with cisplatin. As such, we propose the following algorithm for selecting which patients to start on TEE prophylaxis (**Figure 2**). Balancing slightly higher risk of major bleeding with DOAC compared to LMWH with possible better compliance with an oral medication should be discussed on an individualized basis, since both are FDA approved options. Important future directions of research on this topic will involve methods of minimizing central line use in outpatient chemotherapy regimens and prospective evaluation of the effectiveness of our prophylaxis algorithm on preventing TEE and bleeding risk.

## Conclusions

Patients with advanced germ cell tumors receiving cisplatin-based chemotherapy have a high rate of TEE, which can negatively affect their overall survival. Multiple factors likely increase the risk of TEE in this cohort and can be used to help identify patients who may benefit from primary TEE prophylaxis with LMWH or DOAC. We believe higher utilization of primary TEE prophylaxis in GCT patient starting cisplatin-based chemotherapy would be clinically beneficial and have developed an algorithm to help guide clinical management of these patients.

## Author Contributions

Conception and design, AB, VM, CN, KC, and WA. Acquisition of data, XM. Analysis and interpretation of data, XM and AB. Drafting of the manuscript, XM and MA. Critical revision of the manuscript for important intellectual content, XM, MA, KC, WA, II, VM, CN, and AB. Administrative, technical, or material support, AB. Supervision, AB. All authors contributed to the article and approved the submitted version.

## Funding

For XM, this work was supported in part by the Urology Care Foundation Research Scholar Award Program and Society for Urologic Oncology Fund for Specialized Program of Research Excellence. The content is solely the responsibility of the authors and does not necessarily represent the official views of the American Urological Association or the Urology Care Foundation.

## Conflict of Interest

The authors declare that the research was conducted in the absence of any commercial or financial relationships that could be construed as a potential conflict of interest.

## Publisher’s Note

All claims expressed in this article are solely those of the authors and do not necessarily represent those of their affiliated organizations, or those of the publisher, the editors and the reviewers. Any product that may be evaluated in this article, or claim that may be made by its manufacturer, is not guaranteed or endorsed by the publisher.

## Abbreviations

IGCCCG, International Germ Cell Cancer Collaborative Group; LDH, Lactate dehydrogenase; LMWH, Low-molecular-weight heparin; DOAC, Direct oral anticoagulants; IV, Intravenous therapy; EP, Etoposide, Cisplatin; BEP, Bleomycin, Etoposide, Cisplatin; GemOx, Gemcitabine, Oxaliplatin; TIP, Paclitaxel, Ifosfamide, Mesna, Cisplatin; VIP, Etoposide, Ifosfamide, Cisplatin; VeIP, Vinblastine, Ifosfamide, Mesna, Cisplatin.
